# Isotropic 25-Micron 3D Neuroimaging Using *ex vivo* Microstructural Manganese-Enhanced MRI (MEMRI)

**DOI:** 10.3389/fncir.2018.00110

**Published:** 2018-12-06

**Authors:** Chika Sato, Kazuhiko Sawada, David Wright, Tatsuya Higashi, Ichio Aoki

**Affiliations:** ^1^Department of Molecular Imaging and Theranostics, National Institute of Radiological Sciences, National Institutes for Quantum and Radiological Science and Technology (QST), Chiba, Japan; ^2^Group of Quantum-State Controlled MRI, QST, Chiba, Japan; ^3^Department of Nutrition, Faculty of Medical and Health Sciences, Tsukuba International University, Ibaraki, Japan; ^4^Department of Neuroscience, Central Clinical School, Monash University, Melbourne, VIC, Australia; ^5^Florey Institute of Neuroscience and Mental Health, Parkville, VIC, Australia

**Keywords:** manganese-enhanced MRI, MEMRI, microstructure, neuroimaging, *ex vivo* MEMRI

## Abstract

MRI observations following *in vivo* administration of Mn^2+^ [manganese (Mn)-enhanced MRI, MEMRI] have been used as an excellent morphological and functional MRI tool for *in vivo* preclinical studies. To detect brain three-dimensional (3D) microstructures, we improved the *ex vivo* MEMRI method for mouse brains after* in vivo* Mn administration and obtained high-resolution MRIs using a cryogenic radiofrequency (RF) coil. Male C57BL/6 mice (*n* = 8) were injected with 50 mM MnCl_2_ intravenously and MEMRIs of the brain were acquired *in vivo* after 24 h, followed by perfusion fixation with a 4% paraformaldehyde (PFA) solution. High-resolution 25-μm isotropic MRIs were successfully acquired from the extracted brain tissue and could identify the brain microstructures, especially in the hippocampus [the pyramidal cell layer through CA1–3 and the dentate gyrus (DG) granular layers (GLs)], cell layers of cerebellum, three sub-regions of the deep cerebellar nucleus, and white matter (WM) structures [e.g., the fasciculus retroflexus (fr) and optic tract in the thalamus]. The following technical conditions were also examined: (i) the longitudinal stability of Mn-enhanced *ex vivo* tissue after *in vivo* administration; and (ii) the effects of mixing glutaraldehyde (GA) with the fixative solution for the preservation of *in vivo* MEMRI contrast. Our results indicate that *ex vivo* MEMRI observations made shortly after fixation maintain the contrast observed *in vivo*. This research will be useful for non-destructive whole-brain pathological analysis.

## Introduction

Evaluations of the individual whole-brain structure are important for the analysis of disease mechanisms and therapeutic efficacy assessments (Angenstein et al., 2007; Badea et al., 2007; Sawiak et al., 2009; Kumar et al., 2014; Scholz et al., 2015). Although optical microscopic observations of serial tissue sections are widely used and three-dimensional (3D) reconstructions of the brain have already been reported (Lein et al., 2007; Vandenberghe et al., 2016), sequential thin sectioning of the entire brain and its image acquisition are time-consuming procedures. *In vivo* MRI has the advantage of facilitating functional, metabolic and kinetic observational research. However, it is difficult to increase the spatial resolution because of the problems of involuntary movement in subjects, respiratory and cardiac motion, and limited scanning time because of anesthesia use. In recent animal studies, *ex vivo* micro-imaging techniques using high-field MRI with high-power gradients and sensitive radiofrequency (RF) coil systems have succeeded in obtaining high spatial resolution 3D imaging (3D micro-MRI) of tissue samples (Cleary et al., 2011; Kamsu et al., 2013), facilitating detailed visualization of morphological information (Johnson et al., 2012; Phillips et al., 2015).

Manganese (Mn) is a positive contrast agent and can also be used as a tissue immersion method in *ex vivo* brain samples (Norris et al., 2013; Wu and Zhang, 2016). Tissue immersion methods using Gd-chelate or Mn are helpful for rapid 3D micro-MRI measurements because of the shortening of longitudinal relaxation time (T1) and higher positive contrast. However, in *ex vivo* immersion methods, Gd-chelate or Mn are distributed throughout the brain and bind to cells/tissue in a non-specific manner, and as such, cannot reflect the “functional” information.

Mn^2+^ can mimic Ca^2+^ in many biological systems and accumulates in the living cell through Ca^2+^ channels (Lin and Koretsky, 1997). Thus, MR imaging following *in vivo* Mn administration (Mn-enhanced MRI, MEMRI) has been used for functional and microstructural imaging (Aoki et al., 2004; Yu et al., 2005; Silva et al., 2008; Watanabe et al., 2008; Chan et al., 2014). Although most MEMRI studies have been performed using *in vivo* administration and *in vivo* observation, *ex vivo* observation after *in vivo* Mn chloride (MnCl_2_) administration is also possible and offers improved spatial resolution without motion artifacts. In addition, *ex vivo* MEMRI may reflect not only the anatomical structures, but also the functional information in whole mice brains. However, there is a dearth of research on *ex vivo* microstructural MEMRI, and only a few *ex vivo* MEMRI studies have been reported (Bangasser et al., 2013; Liu et al., 2013). The Mn^2+^ stability in the fixed tissue may be short, and the perfusion fixation process or immersion to a fixative solution may attenuate the contrast (Huang et al., 2009; Norris et al., 2013). Liu et al. (2013) showed that perfusion fixing with a glutaraldehyde (GA) solution preserved the tissue enhancement of *in vivo* Mn^2+^, in the short term (<48 h); however, they did not assess its long-term stability.

To observe the single-layer 3D structure in *ex vivo* mice brains, we improve the spatial resolution of MRI to 25 μm isotropic using *ex vivo* MEMRI following* in vivo* Mn administration. Specifically, we: (i) optimized the longitudinal stability of *ex vivo* Mn enhancement after *in vivo* administration; and (ii) assessed whether the addition of GA to the fixative solution maintains *ex vivo* MEMRI contrast.

## Materials and Methods

### Animals

All animal experiments were approved by the Animal Welfare Committee of the National Institute of Radiological Sciences, Quantum and Radiological Science and Technology (QST), Chiba, Japan (No. 14-1007-5). Seventeen mice (*Mus musculus*, C57BL/6, male, 8−10 weeks old; CLEA Japan Inc., Tokyo, Japan) were housed under a 12-h light/dark cycle with access to food and water *ad libitum*. Room temperature was consistent at 23 ± 1°C.

### Experimental Design

**Figure 1:**
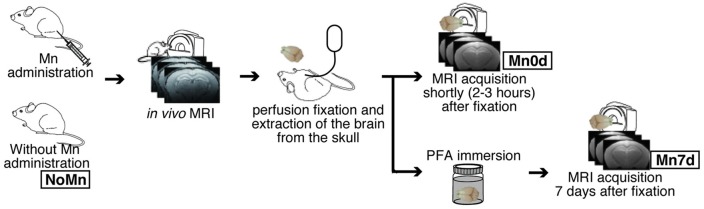
Overview of experimental procedures. Twelve mice were injected with manganese chloride (MnCl_2_) and scanned using a 7-T preclinical MRI 24 h and 7 days later (*in vivo* MRI).

The mice were randomly assigned to one of five groups, having the following characteristics (Figure 1 and Table 1): No Mn administration (“NoMn”; intact mice; *n* = 5; body weight, 27.3 ± 1.0 g); *ex vivo* MEMRI was performed 2–3 h after fixation (“Mn0d”; Mn was infused *in vivo* and MRI measurements were conducted shortly after fixation; *n* = 5; 26.1 ± 0.7 g); and *ex vivo* MEMRI performed 7 days after fixation (“Mn7d”; Mn was infused *in vivo* and the extracted brain was immersed in 4% paraformaldehyde (PFA) solution for 7 days; *n* = 3; 27.9 g ± 0.7 g). The remaining group was divided into two subgroups: *ex vivo* MEMRI perfused by 4% PFA with 1% GA (PFA + 1% GA; *n* = 2; 27.6 ± 0.1 g), and *ex vivo* MEMRI perfused by 4% PFA with 2.5% GA (*n* = 2; 23.7 ± 0.1 g).

**Table 1:** Sample preparation and procedures for each group in Experiment 1.

	*n*	*In vivo* Mn administration	*In vivo* MRI	*Ex vivo* MRI shortly after fixation	*Ex vivo* MRI 7 days after fixation
NoMn	5	−	+	+	+
Mn0d	5	+	+	+	−
Mn7d	3	+	+	−	+
2.5% GA	2	+	+	+	+
1% GA	2	+	+	+	+

To observe the Mn^2+^ preservation in the *ex vivo* brain tissue longitudinally, MnCl_2_ was administered *in vivo* to the mice. T1-weighted (T1W) MRIs were obtained *in vivo* 24 h after the MnCl_2_ administration (*in vivo* MEMRI), and the extracted brain tissue was then observed once again using MRI (*ex vivo* MEMRI). We tested two types of Mn-enhanced samples for the *ex vivo* MEMRI study, i.e., those obtained shortly (Mn0d group) or 7 days after fixation (Mn7d group), to examine the influence of the perfusion fixation and the stability of the Mn in the tissue. Moreover, to evaluate the effects of GA against accumulated Mn in brain tissue, we observed the Mn-enhanced sample using a fixative solution with 1% and 2.5% GA.

### *In vivo* MEMRI

Of the 17 mice, 12 were administered MnCl_2_ (50 mM, osmotic pressure-controlled, 100-mg/kg body weight, 250 μl/h; Sigma-Aldrich, St. Louis, MO, USA) via their tail vein. The *in vivo* MEMRI of the mouse brains was performed using a 7.0 T preclinical MRI (Avance-III, 20-cm bore; Bruker Biospin, Billerica, MA, USA) with a cryogenically cooled RF coil 22 h after Mn administration. Thirteen mice were anesthetized using 2% isoflurane (Mylan Inc., Osaka, Japan) in O_2_ for induction and maintenance, and were fixed in a designated cradle by ear and bite bars. Their respiration and rectal temperatures were monitored during measurements. An *in vivo* T1W 3D image was obtained using the rapid acquisition with relaxation enhancement (RARE) sequence: repetition time (TR)/echo *time* (TE) = 400/8.65 ms (effective TE = 8.65 ms), RARE factor = 2, field of *view* (FOV) = 12 × 8 × 15 mm^3^, matrix = 160 × 108 × 100, voxel size = 75 × 75 × 150 μm^3^, number of *averages* (NA) = 1, and total scan time = 36 min. A RARE sequence with variable repetition time was used for T1 and T2 mapping with the following settings: TR = 200, 400, 800, 1,500, 3,000 and 5,000 ms; TE = 11, 33, 55, 77 and 99 ms; FOV = 19.2 × 19.2 mm^2^; acquisition matrix = 256 × 256; and total scan time = 17 min 26 s.

### Perfusion Fixation and Tissue Preparation

After the *in vivo* MEMRI, the mice were deeply anesthetized using sodium pentobarbital (50 mg/kg body weight, i.p., Somnopentyl, Kyoritsu Seiyaku Corporation, Tokyo, Japan) following 2% isoflurane and transcardial perfusion fixation using saline and fixative solutions. The types of fixative solutions were 4% PFA in 0.1 M phosphate buffered saline (PBS, pH 7.4; Wako Pure Chemical Industries Ltd., Osaka, Japan), and 1% and 2.5% GA (Wako Pure Chemical Industries) in 4% PFA/PBS for the PFA + 1% or 2.5% GA group. The perfusion fixation was initiated 24 h after Mn administration. The mouse brains were carefully removed from the skull and immersed in a fluorine compound (Fomblin, CF_3_O[-CF(CF_3_)CF_2_O-]_x_(-CF_2_O-)_y_CF_3_; Solvay Solexis, NJ, USA) for *ex vivo* MEMRI. For longitudinal observation, the extracted brains using PFA were kept in the fixative solution for 7 days at 4°C and immersed in a fluorine compound. For the experiments of GA addition to the fixative solution, the fluorine compound was gently washed out from the brain samples perfused by PFA/GA using PBS after *ex vivo* MEMRI shortly after fixation. The extracted brains were kept in the PFA/GA fixative solution for 7 days at 4°C, and re-immersed in a fluorine compound for longitudinal observation.

### *Ex vivo* MEMRI

The *ex vivo* images were obtained using the same 7.0-T MRI scanner and coil as for the *in vivo* study. *Ex vivo* T1W 3D images were obtained using RARE and a fast low-angle shot (FLASH) sequence. The parameters of the RARE, T1 and T2 map sequences were identical to those listed in subsection 2.2.1. The FLASH sequence parameters were TR/TE = 200/9 ms, flip angle = 60°, FOV = 7.5 × 10 × 7.5 mm^3^, matrix = 300 × 400 × 300, voxel size = 25 × 25 × 25 μm^3^, and NA = 2. The total scan time was 14 h. The image acquisition was performed in the following order: T1 and T2 mapping, RARE, and FLASH. The entire scanning time was 14 h 53 min.

### Data Analysis

*In vivo* and *ex vivo* MRI data were reconstructed using ParaVision (Version 5.1, Bruker-Biospin, Billerica, MA, USA), imported into Fiji/ImageJ software (Version 1.0, National Institutes of Health), and the contrast was optimized. Signal intensities of T1W images for the longitudinal *ex vivo* MRI were adjusted based on the background signal level. Quantitative T1 and T2 maps were calculated using ParaVision. R1 and R2 values were calculated for a number of regions of interest (ROIs) including the retrosplenial agranular and granular cortices, association cortex, somatosensory cortex and auditory cortex (Bregma −1.82 mm). ROIs were delineated according to the Paxinos mouse brain atlas (Paxinos and Franklin, 2013). The brain volumes were measured using the OsiriX image viewer (Pixmeo, Switzerland). The raw data supporting the conclusions of this manuscript will be made available by the authors, without undue reservation, to any qualified researcher.

### Statistical Analysis

For the statistical analysis, one-way analysis of variance (ANOVA) with the Tukey–Kramer method was implemented for all cases using the “anovan” and “multcompare” functions in MATLAB (R2016b, Mathworks, Natick, MA, USA). The MRI signal intensity profiles were computed using ImageJ software and the Z-scores of the profiles were calculated using a MATLAB software function, “z-score,” calculated as follows:

$$\text{Z = (v}-{\text{v}}_{\text{ave}}{\text{)/SD}}_{\text{profile}}$$

where v is the signal intensity in a single voxel, v_ave_ is the mean value, and SD_profile_ is the standard deviation of the pixel intensity on the profile line. For the R1 and R2 calculations of sample solutions of the contrast agents, linear regression fittings were performed for different concentrations and slope differences were compared statistically using Prism 6 (Version 6.0b, GraphPad Software Inc., La Jolla, CA, USA).

## Results

### *In vivo* and *ex vivo* MEMRI After *in vivo* Mn Administration

**Figure 2:**
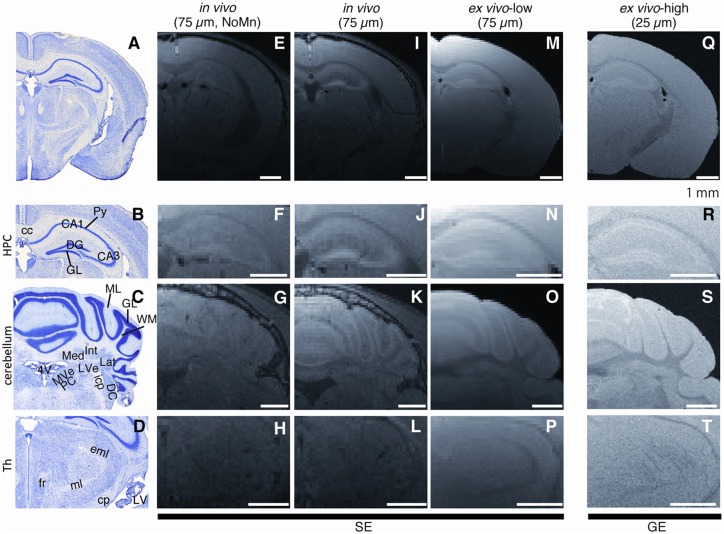
Observable differences between *in vivo* and *ex vivo* Mn-enhanced MRI (MEMRI). **(A,E,I,M,Q)** Typical examples of T1-weighted (T1W) images of *in vivo* and *ex vivo* MEMRI for Mn0d group. (Left to right) Histological from Paxinos and Franklin (2013), *in vivo* without Mn administration (75 μm), *in vivo* with Mn (75 μm), *ex vivo* low resolution (75 μm), *ex vivo* high resolution (25 μm) images. We used 4% paraformaldehyde (PFA) following saline for perfusion fixation. Scale bar: 1 mm. Magnified images in the hippocampus (**B,F,J,N,R**; HPC, Bregma −2.18 mm), cerebellum (**C,G,K,O,S**; Bregma −6.00 mm), and thalamus (**D,H,L,P,T**; Th, Bregma −2.46 mm). Single-averaging data were presented for **(Q–T)** due to the artifact. cc, corpus callosum; CA1, Ammon’s horn 1; CA3, Ammon’s horn 3; DG, dentate gyrus; Py, pyramidal cell layer; GCL, granular cell layer; ML, molecular cell layer; WM, white matter; Med, medial cerebellar nucleus; Int, interposed cerebellar nucleus; Lat, lateral cerebellar nucleus; LVe, lateral vestibular nucleus; MVePC, medial vestibular nucleus, parvicellular part; Pr, prepostius nucleus; DC, dorsal cochlear nucleus; icp, inferior cerebellar peduncle; 4V, 4th ventricle; fr, fasciculus retroflexus; ml, medial lemniscus; eml, external medullary lamina; cp, cerebral peduncle; LV, lateral ventricle; SE, spin echo image; and GE, gradient echo image.

The stability of Mn accumulation following fixation (*in vivo* vs. *ex vivo*) was compared using 75-μm in-plane resolution (Figure 2). Shortly after the perfusion fixation, the extracted Mn-enhanced brain tissue (Figure 2M, MN0d group, *ex vivo* MEMRI) showed a contrast similar to that for the *in vivo* MEMRI (Figure 2I) while attenuating the signal intensity and contrast slightly. The hippocampal laminar structure in the extracted tissue (*ex vivo* MEMRI) exhibited positive signal enhancement (Figure 2N), similar to that for the *in vivo* MEMRI (Figure 2J). In the cerebellum, the *ex vivo* MEMRI showed positive enhancement strongly in the granular cell layer (GCL) and moderately positive enhancement in the molecular layer (Figure 2O), also similar to that for the *in vivo* cases (Figure 2K).

In the 25-μm high-spatial-resolution 3D measurement for the *ex vivo* MEMRI (Figure 2Q), we could observe the brain structures more clearly, especially in the cell layers of the hippocampus and cerebellum (Figures 2R,S), when compared to either the 75-μm spatial resolution *ex vivo* MRI or *in vivo* MRI. For the 25-μm 3D *ex vivo* MEMRI, the pyramidal cell layer through CA1 to CA3 and the dentate gyrus (DG) granular layers (GLs) in the hippocampus were more clearly defined than in the 75-μm 3D *ex vivo* MEMRI (Figure 2R). In the cerebellum, three subregions of the deep cerebellar nucleus, i.e., the medial, interposed and lateral nuclei, were distinctly observed by increasing the spatial resolution with respect to the surrounding white matter (WM; Figure 2S). The WM structures (e.g., the fasciculus retroflexus (fr) and optic tract in the thalamus; Figure 2T) were also delineated more clearly compared to the *in vivo* and 75-μm 3D *ex vivo* MEMRI. Thus, the *ex vivo* MEMRI method allowed microstructure brain imaging with a contrast similar to that for *in vivo* MEMRI.

**Figure 3:**
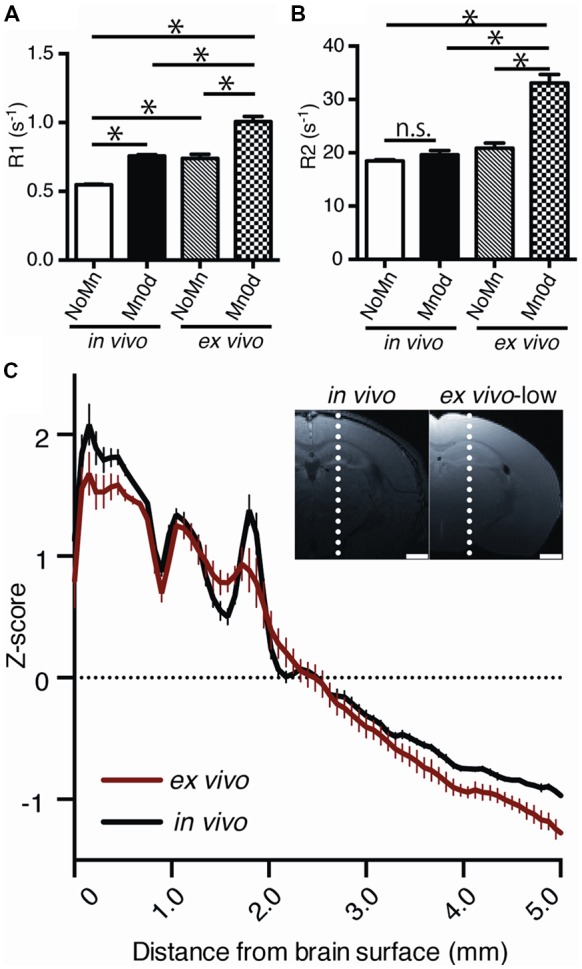
Comparison of relaxation rate (R1, R2) and contrast between *in vivo* and *ex vivo* MEMRI. R1 **(A)** and R2 **(B)** at the cerebral cortex between *in vivo* and *ex vivo* (NoMn: *n* = 5; Mn0d; *n* = 5). R1 and R2 were calculated as a mean value of the cortex between the right and left hemispheres. The z-scores of signal profile of the brain **(C)**, which are indicated by the broken lines in the inset image, were calculated from the T1-weighted (T1W) image; the averages of z-scores for the *in vivo* (black, *n* = 5) and *ex vivo* (red, *n* = 5) cases are presented. NoMn, No Mn administration; Mn0d, Mn administered 0 d after fixation. The error bar shows the standard error of the mean (SEM). *P < 0.05.

To assess the Mn stability in the *ex vivo* tissue quantitatively, the R1 and R2 in the cerebral cortex were compared among administration groups (Mn0d and NoMn) and MRI procedures (*in vivo* and *ex vivo*). In both *in vivo* and *ex vivo* MRI measurements, Mn0d groups exhibited significantly higher R1 in comparison to NoMn administration groups (Figure 3A, *F*_(3,8)_ = 121.6, *P* = 0.0002). These results suggest that Mn accumulated in the brain and enhanced R1 in the same manner as reported in a previous *in vivo* study (Silva et al., 2004). In both Mn0d and NoMn groups, the *ex vivo* groups exhibited significantly higher R1 than that of the *in vivo* groups (Figure 3A; Mn0d, *P* < 0.0001, NoMn; *P* < 0.0001). Interestingly, the R2 of the *ex vivo* Mn0d was significantly higher than that of the *ex vivo* NoMn group (Figure 3B; *F*_(3,8)_ = 49.55, *P* < 0.0001), while there was no significant difference in the R2 between NoMn and Mn0d *in vivo* (*P* = 0.7452). To evaluate the Mn contrasts in the brain structures, we plotted the normalized signal profile (*z*-score) in the T1W image and compared the *in vivo* and *ex vivo* cases (Figure 3C). The profile analysis indicated that the contrast of the cortical layer structures in *ex vivo* MRI was maintained in comparison to that of *in vivo* MRI, although the *ex vivo* MRI showed a slightly broader distribution (Figure 3C). Based on those results, we speculate that the Mn in the *ex vivo* sample was bound to the brain tissue and induced R1 and R2 enhancement through the effect of crosslinking of the PFA fixation. The large R2 enhancement *ex vivo* will induce signal reduction in the Mn-enhanced tissue, such as WM, in high-field MRI systems.

### Longitudinal *ex vivo* Tissue Observation of Mn Preservation After Fixation

**Figure 4:**
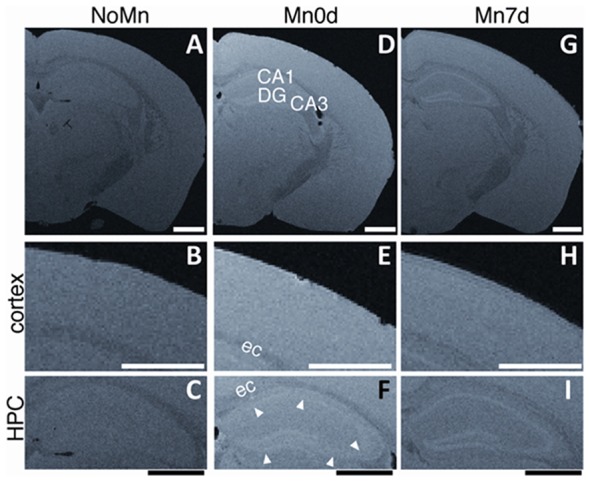
Contrast alterations of *ex vivo* MEMRI after fixation. Differences between Mn-enhanced samples scanned shortly (Mn0d, **D–F**, as same as Figure 2) and 7 days (Mn7d, **G–I**) after perfusion fixation, and an example of *ex vivo* MEMRI without Mn administration (NoMn, shortly after perfusion fixation, **A–C**). Magnified images of the cortex **(B,E,F)** and hippocampus (HPC, **C,F,I**). The white arrowheads in **(F)** show the GCL in the DG and the pyramidal cell layer of the Ammon’s horn. CA1, Ammon’s horn 1; CA3, Ammon’s horn 3; DG, dentate gyrus; cc, corpus callosum. Scale bar: 1 mm.

To examine the longitudinal stability of the Mn in the tissue, we compared *ex vivo* Mn-enhanced brain samples for Mn0d and Mn7d specimens (Figure 4). For the Mn7d group (Figures 4G–I; 7 days after the fixation), the signal intensity and contrast of both the cerebral cortex and hippocampus were attenuated in comparison to the Mn0d group (Figures 4D–F; shortly after the fixation), especially throughout the cerebral cortex (Figures 4E,H). These results indicate that the accumulated Mn in the brain tissue gradually leaked into the PFA fixative solution after fixation. Interestingly, the contrast in the hippocampal laminar structure (pyramidal cell layer in Ammon’s horn and GCL in the DG) was preserved well in the Mn7d sample (Figure 4I). This result indicates that most of the accumulated Mn in *ex vivo* brain-tissue samples disappeared within 7 days due to the 4% PFA immersion, and remained in a part of the hippocampal structure. In summary, although the *in vivo* administered Mn-enhanced tissue gradually lost contrast after fixation, the contrast of laminar structures was maintained for at least 15 h (acquisition time for high resolution 3D imaging) after PFA fixation.

**Figure 5:**
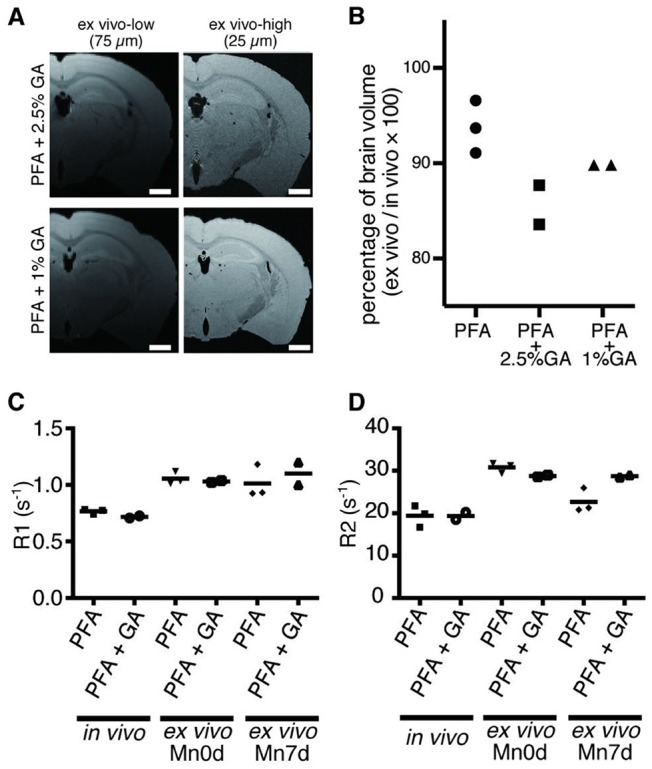
Effects of GA fixation. **(A)** The T1W images obtained through *ex vivo* MEMRI are shown. (Left to right) Low spatial resolution (75 μm) and high spatial resolution (25 μm) shortly after fixation. The *ex vivo* samples were fixated using 2.5% GA in PFA (top), and 1% GA in PFA (bottom). Scale bar: 1 mm. **(B)** The graph shows the ratio of brain volumes of *ex vivo* and *in vivo* samples. **(C,D)** Longitudinal comparison of R1 **(C)** and **(D)** R2 between samples prepared using 4% PFA alone and 1% GA in PFA. PFA, paraformaldehyde; GA, glutaraldehyde.

It has been reported that perfusion fixation with PFA and GA can preserve the Mn in the extracted brain tissue after *in vivo* administration (Liu et al., 2013). Thus, we examined the perfusion fixation method using GA and PFA for its preservation capability of Mn in the *ex vivo* tissue and its deformation. The results showed that there was no difference in the tissue contrast between the samples with and without GA, both shortly (Figure 5A) and 7 days after the fixation (Supplementary Figure S1). Moreover, the tissue samples that were fixed with GA showed volume reduction in the brain tissue (Figure 5B). Therefore, we found that the 4% PFA without GA can maintain the intact brain morphology with the Mn enhancement.

## Discussion

### *Ex vivo* Tissue Observation Using MRI for Neuroscience

Numerous studies have reported the morphological phenotypes of whole-brain structures in animal models, such as transgenic mice or in mice following treatment (Angenstein et al., 2007; Badea et al., 2007; Sawiak et al., 2009; Kumar et al., 2014; Scholz et al., 2015). Histological observations using an optical or electron microscope are the standard methods for assessing tissue at the cellular level in both clinical tests and preclinical studies. Recently, technology for 3D reconstruction using sequential tissue sections has been established, providing whole-brain 3D data sets (Lein et al., 2007; Vandenberghe et al., 2016). However, the preparation of sequential tissue sections for each individual animal remains a challenging task because of the longer preparation time, difficult sectioning, and morphology distortion. Recently, the SNR for MRI has been improved using a high magnetic field, low-noise cryogenic coil, and rapid 3D acquisition for signal averaging. In comparison to *in vivo* animal observation, the measurement of an *ex vivo* tissue sample can permit longer acquisition times leading to higher SNR and facilitating higher spatial resolution 3D images. Although *ex vivo* MRI has lower spatial resolution in comparison to the optical microscope, a 3D microstructure at a spatial resolution of 20–50 μm can be easily acquired in a nondestructive manner with negligible morphological distortion (Cleary et al., 2011; Johnson et al., 2012). We attempted to establish an *ex vivo* microstructural MRI technique using high-field MRI, a high-SNR cryogenically cooled coil and “MRI staining” with contrast agents. Our results showed that the Mn contrast agent, together with 25-μm isotropic 3D spatial resolution, clearly distinguished some laminar structures such as CA1, CA3 and DG in the hippocampus *ex vivo* samples (Figures 2, 2, 4). Contrast-enhanced microstructural *ex vivo* MRI, such as that demonstrated in the present study, will contribute to the assessment of cell-layer level alteration for some diseases, such as the visualization of small amyloid plaques in Alzheimer’s disease (Dhenain et al., 2006; Vandenberghe et al., 2016).

### Stability and Preservation of Mn Inside Tissue

Although the systemic intravenous administration of MnCl_2_ provides an excellent layer contrast in the central nervous system *in vivo* (Aoki et al., 2004; Silva et al., 2008; Watanabe et al., 2008), the longitudinal stability of the Mn-enhanced contrast after fixation has not been well investigated. In the present study, the Mn accumulation in the brain after *in vivo* Mn administration was well preserved in the cortical layer after perfusion fixation using 4% PFA (Figure 2) and the observed contrast on *ex vivo* MEMRI is consistent with results from previous *in vivo* studies (Aoki et al., 2004; Silva et al., 2008). The preservation of Mn in *ex vivo* tissue using GA with PFA fixative has been attempted previously (Liu et al., 2013). Considering the previous report and our experiments using PFA with 1 or 2.5% GA fixation (Figure 5), GA with PFA fixation cannot preserve Mn for a long duration and has the risk of morphological distortion. Our finding that after PFA fixation the preserved Mn gradually leaked from the tissue in a time-dependent manner indicates that the contrast for *ex vivo* MEMRI is unstable (Figure 4). Therefore, fixation using 4% PFA with rapid acquisition within 1 day after extraction is an effective method for preserving the accumulated Mn without brain volume alteration for *ex vivo* tissue MRI. A pioneering study reported that *ex vivo* MEMRI using 4% PFA could detect differences in the neural response between acute novel stressors *in vivo* (Bangasser et al., 2013). Future work needs to develop a more precise and stable method to preserve the accumulated Mn without morphological alteration in *ex vivo* tissue.

### Brain Tissue Contrast of *ex vivo* MEMRI

High isotropic spatial resolution (25 μm) MEMRI detected brain microstructures such as the WM structures, which, to our knowledge, have never been observed in previous MEMRI studies (Figure 2). MEMRI allows the acquisition of functional (Lin and Koretsky, 1997; Aoki et al., 2002; Yu et al., 2005; Chuang et al., 2009; Radecki et al., 2014), neural tract tracing (Pautler et al., 1998; Majid et al., 2014), or morphological (Aoki et al., 2004; Silva et al., 2008; Watanabe et al., 2008) imaging *in vivo*. Therefore, the combination of *in vivo* MEMRI and high-resolution 3D micro-MRI may enable us to analyze not only the brain microstructure, but also its relevant functions (Bangasser et al., 2013) at a cell-layer level. Note that there is a potential risk of artifacts when performing *ex-vivo* MEMRI with samples contained in fluorine oil. We observed truncation ringing artifacts in one sample that resembled the cortical layers and may be misinterpreted as such. It is not clear what caused this artifact although we suspect that it was possibly due to the susceptibility difference between the brain and fluorine oil. However, the images shown here were acquired in a subsequent sample using identical imaging parameters and was free of any artifact. Future studies are needed to more thoroughly investigate the source of these artifacts.

Surprisingly, the enhanced contrast of the hippocampal laminar structure (pyramidal and GCLs, predominantly) remained even 7 days after perfusion fixation with 4% PFA (Figure 4), despite the disappearance of Mn contrast in other brain regions. The finding suggests at least two possibilities. One is that the hippocampal pyramidal and granular cells passively reached a higher Mn concentration in comparison to the other brain regions. It has been reported that the hippocampal cell layers in a Mn-immersed brain sample (the brain was immersed in MnCl_2_ solution after fixation) exhibited positive contrast (Huang et al., 2009; Liu et al., 2013). In addition, the layers have a high density of Ca^2+^ binding sites on the cell membrane (Hinds et al., 2003). The other possibility is that the hippocampal pyramidal cell layer and GCL actively accumulate high Mn concentration *in vivo*.

## Conclusion

A methodology for *ex vivo* MEMRI observation after *in vivo* Mn administration was investigated for high-resolution 3D brain observation. After *in vivo* Mn administration, the contrast in *ex vivo* MEMRI within 24 h of fixation was similar to the *in vivo* one and lost the Mn enhancement 7 days after fixation, except for pyramidal and GCLs in the hippocampus. The layer structures of particular regions of the mouse brains could be visualized in 3D isotropic 25-μm spatial resolution. Further, a 4% PFA solution with 2.5% or 1% GA could not completely retain the Mn in the fixed tissue for 7 days and altered the brain volume. To increase the spatial resolution and SNR of the functional MEMRI, an *ex vivo* approach with a method for improved Mn preservation in the fixed tissue is required. We expect that the methods developed in this work will help to improve morphological and functional 3D imaging of brain.

## Author Contributions

IA and CS contributed to the conception and design of the study. CS and DW performed the imaging. CS and KS performed the data and statistical analysis. CS wrote the first draft of the manuscript. IA, KS, DW and TH wrote sections of the manuscript. All authors contributed to manuscript revision, read and approved the submitted version.

## Conflict of Interest Statement

The authors declare that the research was conducted in the absence of any commercial or financial relationships that could be construed as a potential conflict of interest. The reviewer AI and handling editor declared their shared affiliation at time of review.
