# Supplementary material for: Isotropic 25-Micron 3D Neuroimaging Using ex vivo Microstructural Manganese-Enhanced MRI (MEMRI)
Source: Front Neural Circuits. 2018 Dec 6;12:110. doi: 10.3389/fncir.2018.00110 (PMC6291442; doi:10.3389/fncir.2018.00110)
Supplement: Supplementary file 1 [file Image_1.pdf]

Isotropic 25-micron 3D neuroimaging using *ex vivo* microstructural manganese-enhanced MRI (MEMRI)

Chika Sato, Kazuhiko Sawada, David K. Wright, Tatsuya Higashi, Ichio Aoki

Supplementary Figures and Figure Legends

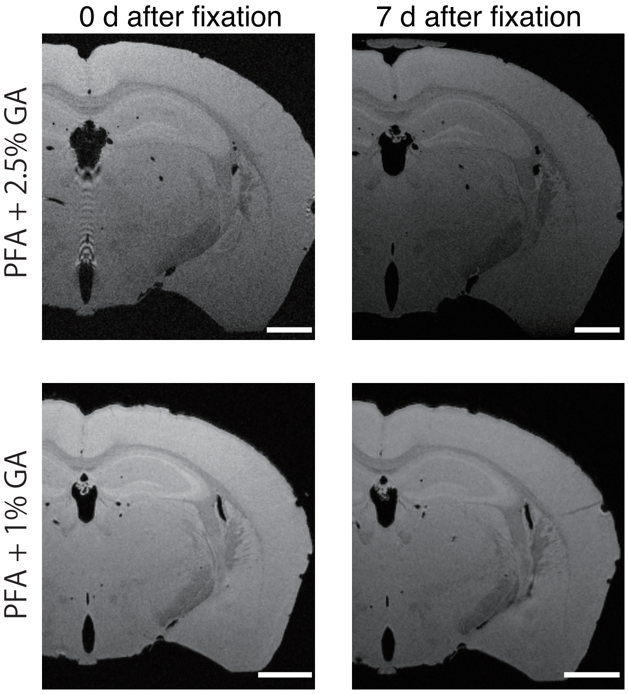

Supplementary Figure S1. Longitudinal Observation of preserved Mn in *ex vivo* brain with different fixative solution. The T1-weighted images obtained through *ex vivo* Mn-enhanced MRI shortly after fixation (left column, as same as Figure 1C) and 7 days after fixation (right column) were shown. We used 4% PFA including 2.5% GA (top), or in 1% GA (bottom) as fixative solution. PFA: parafolmaldehyde, GA: glutaraldehyde. Scale bar: 1 mm.
